# TNF-Receptor-1 inhibition reduces liver steatosis, hepatocellular injury and fibrosis in NAFLD mice

**DOI:** 10.1038/s41419-020-2411-6

**Published:** 2020-03-31

**Authors:** Franziska Wandrer, Stephanie Liebig, Silke Marhenke, Arndt Vogel, Katharina John, Michael P. Manns, Andreas Teufel, Timo Itzel, Thomas Longerich, Olaf Maier, Roman Fischer, Roland E. Kontermann, Klaus Pfizenmaier, Klaus Schulze-Osthoff, Heike Bantel

**Affiliations:** 1 Department of Gastroenterology, Hepatology and Endocrinology, Hannover Medical School, Hannover, Germany; 2 Department of Medicine II, Division of Hepatology, Medical Faculty Mannheim, University of Heidelberg, Mannheim, Germany; 3 Institute of Pathology, University of Heidelberg, Heidelberg, Germany; 4 Institute of Cell Biology and Immunology, University of Stuttgart, Stuttgart, Germany; 5 Interfaculty Institute of Biochemistry, University of Tübingen, Tübingen, Germany; 6 German Cancer Consortium (DKTK) and German Cancer Research Center (DKFZ), Heidelberg, Germany

**Keywords:** Fatty acids, Metabolic syndrome

## Abstract

Non-alcoholic fatty liver disease (NAFLD) shows an increasing prevalence and is associated with the development of liver fibrosis and cirrhosis as the major risk factors of liver-related mortality in this disease. The therapeutic possibilities are limited and restricted to life style intervention, since specific drugs for NAFLD are unavailable so far. TNFα has been implicated as a major pathogenic driver of NAFLD. TNFα-mediated liver injury occurs mainly via TNF-receptor-1 (TNFR1) signaling, whereas TNFR2 mediates protective pathways. In this study, we analyzed the therapeutic effects of a novel antibody, which selectively inhibits TNFR1 while retaining protective TNFR2 signaling in a high-fat diet (HFD) mouse model of NAFLD. Mice were fed with HFD for 32 weeks and treated with anti-TNFR1-antibody or control-antibody for the last 8 weeks. We then investigated the mechanisms of TNFR1 inhibition on liver steatosis, inflammatory liver injury, insulin resistance and fibrosis. Compared to control-antibody treatment, TNFR1 inhibition significantly reduced liver steatosis and triglyceride content, which was accompanied by reduced expression and activation of the transcription factor SREBP1 and downstream target genes of lipogenesis. Furthermore, inhibition of TNFR1 resulted in reduced activation of the MAP kinase MKK7 and its downstream target JNK, which was associated with significant improvement of insulin resistance. Apoptotic liver injury, NAFLD activity and alanine aminotransferase (ALT) levels, as well as liver fibrosis significantly decreased by anti-TNFR1 compared to control-antibody treatment. Thus, our results suggest selective TNFR1 inhibition as a promising approach for NAFLD treatment.

## Introduction

Non-alcoholic fatty liver disease (NAFLD) represents one of the most common causes of chronic liver diseases, ranging from simple steatosis (non-alcoholic fatty liver; NAFL) to non-alcoholic steatohepatitis (NASH)^1^. NASH is histologically characterized by the presence of liver steatosis and evidence of liver injury which can result in the development of liver fibrosis/cirrhosis^2^. Patients with NASH and fibrosis are at risk of developing end-stage liver disease and hepatocellular carcinoma, as well as extrahepatic complications, such as cardiovascular diseases or non-hepatic malignancies^3^. There is no approved therapy so far available for NASH patients, and lifestyle modification remains the only treatment option. Therefore, the identification of novel targets and therapies is urgently needed.

Activation of pro-inflammatory cytokines, such as TNFα, in adipose and liver tissues has been implicated to play an important role in the pathogenesis and disease progression of NAFLD^4–6^. Higher TNFα serum levels which correlate with insulin resistance were reported for NASH patients compared to patients with simple steatosis^7–9^. Moreover, in liver tissues of NASH patients enhanced TNFα and TNF-receptor (TNFR) 1 expression correlated with disease activity and fibrosis stages^4^. Vice versa, in various diet-induced or genetic NAFLD models, TNFα-deficient or TNFR-deficient mice showed improved insulin sensitivity and less pronounced liver steatosis and fibrosis^10–12^.

TNFα is a pleiotropic cytokine which regulates pathways involved in inflammation, cell metabolism and tissue homeostasis. TNFα signal transduction is mediated by TNFR1 and TNFR2. Whereas TNFR1 is ubiquitously expressed, TNFR2 expression is more restricted to immune cells, endothelial cells and neurons^13^. Binding of TNFα to TNFR1 leads to protein complex formation with cytoplasmic adapter proteins and the TNF receptor-associated factor (TRAF) 2 or the receptor-interacting protein-1, which is followed by the activation of mitogen-activated protein (MAP) kinases including c-Jun-N-terminal kinase (JNK) or the nuclear factor κB (NFκB) pathway^8,14^. In addition, the TNFα/TNFR1 complex can interact with the adapter protein FADD which leads to the recruitment and activation of initiator caspase-8^14,15^. Activated caspase-8 activates downstream effector caspases such as caspase-3, caspase-6, or caspase-7 which cleave cellular substrates and thereby induce the apoptotic cell death^16–19^. TNFR1-mediated pathways create a pro-inflammatory microenvironment which not only plays a role for insulin resistance, liver injury, fibrogenesis and thus for NAFLD progression, but also critically contributes to the development of obesity-related hepatocellular carcinoma^8,20–22^.

Whereas TNFR1 plays a role in deleterious pathways leading to inflammation, metabolic alterations and cell death, TNFR2 is mainly involved in protective pathways of regeneration, cell survival and immune response regulation^13,23–25^. Because of the importance of TNFα in NAFLD and the distinct roles of the two TNFRs, therapeutic interventions should lead to NAFLD regression, but avoid side effects, such as infections, which are frequently observed with conventional TNFα antagonists. Selective inhibition of TNFR1 and maintenance of beneficial effects of TNFR2-mediated protective pathways might therefore represent a promising treatment strategy for NAFLD.

In this study, we investigated the therapeutic potential of a novel antibody specific for human TNFR1. To this end, we employed a NAFLD model using humanized TNFR1 knock-in mice, in which the extracellular domain of murine TNFR1 was exchanged with the human TNFR1 counterpart^25^. We analyzed the effect of selective TNFR1 inhibition on liver steatosis, liver injury, insulin resistance and fibrosis and unraveled the underlying pathways. Our data show that TNFR1 inhibition results in a significant improvement of liver steatosis and insulin resistance, as well as of liver injury and fibrosis. Selective TNFR1 inhibition might therefore represent a promising treatment strategy in NAFLD.

## Methods

### Treatment of mice

Male humanized TNFR1 knock-in (huTNFR1-k/i) mice^25^ in a C57/BL6J background (age 6–12 weeks) were used. Mice received a high-fat diet (HFD) consisting of 60% kcal of fat (Altromin, Lage, Germany), supplemented with 42 g/L sugar (55% fructose, 45% sucrose) in the drinking water^26^ for 24 weeks, before therapeutic intervention was initiated by the administration (i.p.) of an anti-humanTNFR1 (IgG1) antibody (20 mg/kg body weight, 2×/week, *n* = 7)^27–29^ for further 8 weeks. The anti-EGFR antibody cetuximab (IgG1; Merck KGaA; Darmstadt, Germany), which is not cross-reactive with mouse EGFR, served as negative control (*n* = 6). Mice were randomly distributed to the different treatment groups. Mice of both groups revealed no significant difference in age. At the end of treatment body weight of the mice was assessed, and blood samples were taken for analyses of ALT levels and metabolic parameters. After the mice were sacrificed, liver weight was determined. Liver tissues from the differentially treated mice were analyzed for liver steatosis, caspase activation and fibrosis. In addition, the percentage of steatosis, as well as the NAFLD activity score (NAS), which considers steatosis, lobular inflammation and hepatocellular ballooning^30^, were assessed in liver sections by a pathologist blinded to the treatment groups. Animal care and treatment were performed according to the federal guidelines and approved by university and state authorities.

### Analyses of blood and liver tissue samples

Plasma insulin levels were measured by enzyme-linked immunosorbent assay (ELISA) according to manufacturer’s instructions (Crystal Chem, Zaandam, Netherlands). The HOMA-IR (homeostasis model assessment of insulin resistance) index was calculated as previously described^31,32^. For triglyceride quantification, liver tissue lysates were prepared with Ultra-Turrax® homogenizer in the assay diluent reagent provided by the manufacturer of the colorimetric enzyme test (Cayman Chemical, Ann Arbor, MI, USA). In addition, Oil Red O staining of frozen liver sections was performed according to the manufacturer’s protocol (Sigma-Aldrich, St. Louis, MO, USA). Fibrosis was detected by Sirius Red staining of paraffin-embedded liver sections according to the manufacturer’s protocol (Sigma-Aldrich) and quantified by analyzing four microscopic fields at a 100-fold magnification^33^.

Immunohistochemical detection of activated caspase-3 was performed in paraffin-embedded liver sections as described^34,35^. Briefly, liver sections were incubated with an antibody against activated caspase-3 (Cell Signaling, Danvers, MA, USA) for one hour following antigen retrieval and blocking of endogenous peroxidase. After repeated washings, sections were treated with biotinylated goat anti-rabbit antibody for 30 min and then covered with avidin-biotin complex reagent containing horseradish peroxidase (VECTASTAIN ABC Kit, Vector Laboratories, Burlingame, CA, USA) for one hour. The percentage of cells positive for activated caspase-3 was assessed by analyzing four microscopic fields at a 400-fold magnification.

Immunoblotting was performed as described^36,37^. Frozen liver tissue was homogenized by Ultra-Turrax® in RIPA buffer containing protease (Roche, Basel, Switzerland) and phosphatase inhibitors (AppliChem, Darmstadt, Germany) and centrifuged for 10 min at 16,000×*g*. Proteins from liver tissue lysates were separated under reducing conditions on 10%-SDS-polyacrylamide gels. Antibodies against p-mTOR (Ser2448; #5536), mTOR (#2983), FAS (#3180), SCD1 (#2794), JNK (#9252), p-JNK (Thr183/Tyr185; #9255) and p-IRS1 (Ser307; #2381) were purchased from Cell Signaling. Anti-p-MKK7 (Ser271; OAAF05547) and anti-SREBP1 (MA5-16124) antibodies were obtained from Biozol Diagnostica (Eching, Germany) and Thermo Fisher Scientific (Waltham, MA, USA). MKK7 antibody (ADI-905-655-100) was obtained from Enzo Life Science (Farmingdale, NY, USA). Anti-actin (sc-1615; Santa Cruz, Dallas, TX, USA) or anti-vinculin antibody (#13901; Cell Signaling) served as control. Densitometric analyses comparing phosphorylated to non-phosphorylated proteins were performed by using Image J software and normalized to actin^38^.

### Statistical analyses

Statistical analyses were performed by using GraphPad Prism 5.0 software (GraphPad Software Inc., La Jolla, CA, USA). Data are presented as means ± standard error of the mean (SEM). The data obtained from the differentially treated mice were compared by using the Mann–Whitney’s *U* test. A *P* value < 0.05 was considered significant.

## Results

### Anti-TNFR1 antibody treatment reduces liver steatosis in NAFLD mice

**Fig. 1:**
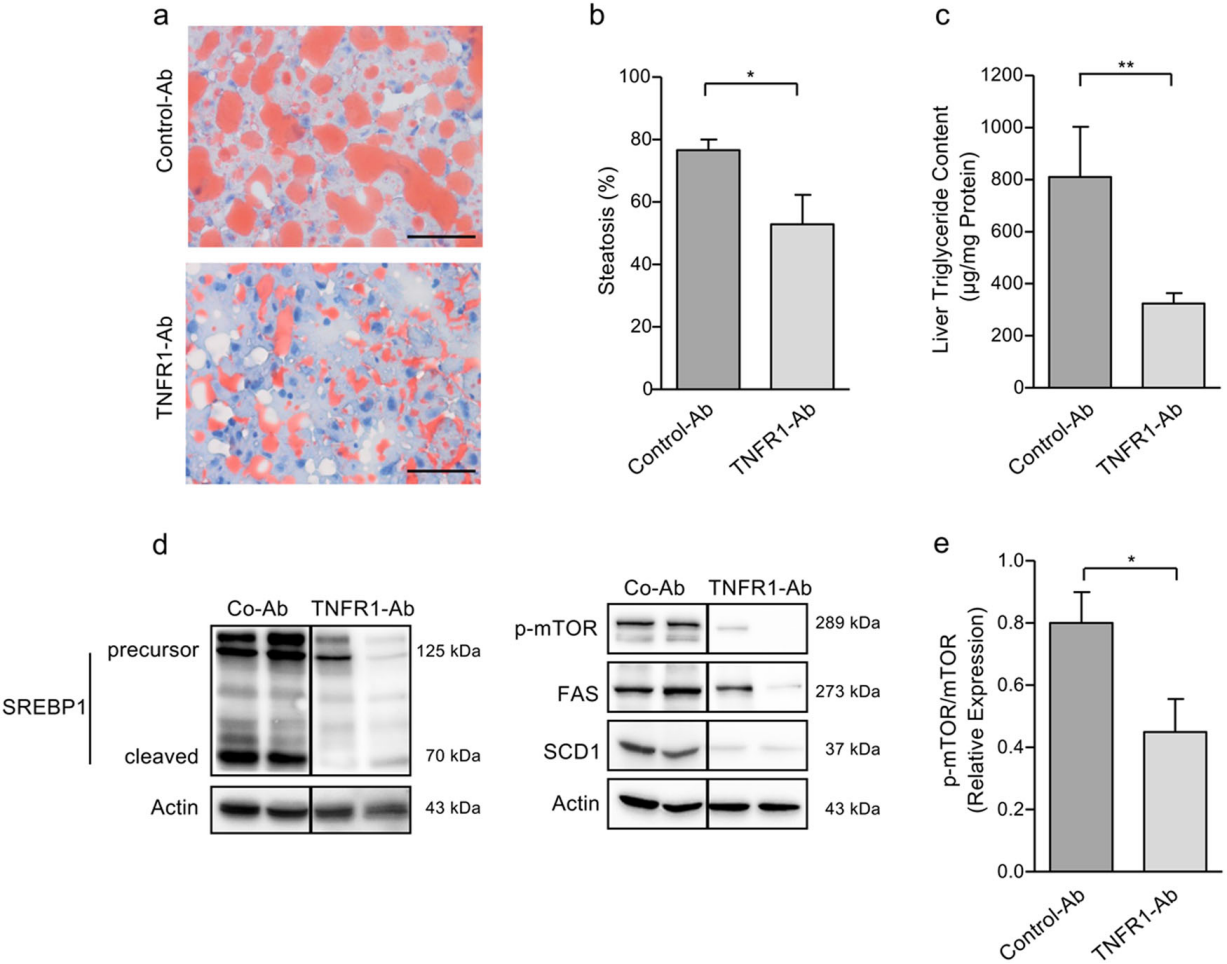
Reduction of liver steatosis by TNFR1 inhibition in NAFLD mice. **a** NAFLD mice treated with anti-TNFR1-Ab for 8 weeks revealed decreased liver steatosis, detected by Oil Red O staining, compared to mice treated with control-Ab (scale bars: 50 µm). Representative results of anti-TNFR1-Ab-treated (*n* = 7) and control-Ab-treated (*n* = 6) mice are shown. **b** Liver steatosis, as semi-quantitatively assessed by pathologist, was significantly decreased in NAFLD mice treated with anti-TNFR1-Ab (*n* = 7) compared to control-Ab (*n* = 6) treatment. **c** Compared to control-Ab administration (*n* = 6), TNFR1-Ab treatment (*n* = 7) resulted in a significant decrease of liver triglyceride content. **d** Western blot analysis of liver extracts from two representative mice treated with either anti-TNFR1-Ab or control-Ab. Mice treated with TNFR1-Ab revealed ameliorated mTOR activation (p-mTOR), as well as decreased expression and cleavage-mediated activation of transcription factor SREBP1 compared to control-Ab-treated mice. Accordingly, expression of SREBP1-regulated target genes of lipogenesis, i.e., fatty acid synthase (FAS) and stearoyl-CoA desaturase-1 (SCD1) was decreased in liver tissues from TNFR1-Ab-treated compared to control-Ab-treated mice. All images were derived from the same blots; the vertical lines indicate juxtaposition of lanes non-adjacent within the same blot. **e** Densitometric analyses revealed a significantly higher ratio of phosphorylated compared to non-phosphorylated version of mTOR in control-treated vs. TNFR1-Ab-treated mice (*n* = 6 per group). All proteins were detected under the same blotting conditions using actin as loading control. *p < 0.05; **p < 0.01.

Since TNFR1 has been implicated to play a major role in NAFLD-associated liver injury, we analyzed potential therapeutic effects of an antagonistic huTNFR1-selective antibody^27–29^. We employed humanized TNFR1 knock-in mice^25^, in which responses of endogenous mouse TNFα to the transgenic huTNFR1 can be modulated by administration of the anti-huTNFR1-selective antibody. The mice were subjected to a 24-week HFD protocol and then treated for additional 8 weeks under HFD with anti-TNFR1-Ab (*n* = 7) or control-Ab (*n* = 6). Treatment of NAFLD mice with anti-TNFR1 significantly reduced liver steatosis as compared to control-Ab treatment (52.9 ± 9.4% vs. 76.7 ± 3.3%, *p* < 0.05; Fig. 1a, b). This was accompanied by a significant decline of liver triglyceride content (324.4 ± 39.7 µg/mg protein vs. 810.2 ± 193.8 µg/mg protein, *p* < 0.01; Fig. 1c).

Since de novo lipogenesis plays an important role in the pathogenesis and disease progression of NAFLD, we analyzed the expression and activation of sterol regulatory element binding protein-1 (SREBP1), a master transcription factor of lipogenesis with SREBP1c as the main isoform expressed in the liver. SREBP1 is synthesized and expressed in the endoplasmic reticulum as a membrane-bound precursor, which is activated by proteolytic cleaving, thereby allowing its nuclear translocation^39^. We found enhanced expression and activation of SREBP1 in liver tissues from control NAFLD mice, which was strongly reduced in mice treated with anti-TNFR1 antibody (Fig. 1d).

Both expression and activation of SREBP1 requires an active mTORC1 pathway, which has been implicated in lipogenesis and NAFLD^40,41^. We therefore analyzed the phosphorylation and activation of mTOR as a potential upstream regulator of SREBP1. Compared to control-antibody treated mice, mTOR activation, as assessed by its phosphorylation at Ser2448, was reduced in liver tissues from mice treated with the anti-TNFR1 antibody (Fig. 1d), while no difference in the basal mTOR expression was detectable (data not shown). Significantly decreased mTOR activation (*p* < 0.05) was confirmed by densitometric analyses comparing the relative expression of phosphorylated to non-phosphorylated mTOR in mouse livers (Fig. 1e). In line with this observation, expression of SREBP1 target genes, i.e., fatty acid synthase (FAS) and stearoyl-CoA desaturase-1 (SCD1), was decreased in liver tissues from anti-TNFR1-treated compared to control-Ab-treated mice (Fig. 1d). These data indicate that TNFR1 inhibition results in reduced mTOR-mediated SREBP1 activation and thus decreases lipogenesis and liver steatosis.

### Inhibition of TNFR1 reduces liver injury and fibrosis in NAFLD mice

**Fig. 2:**
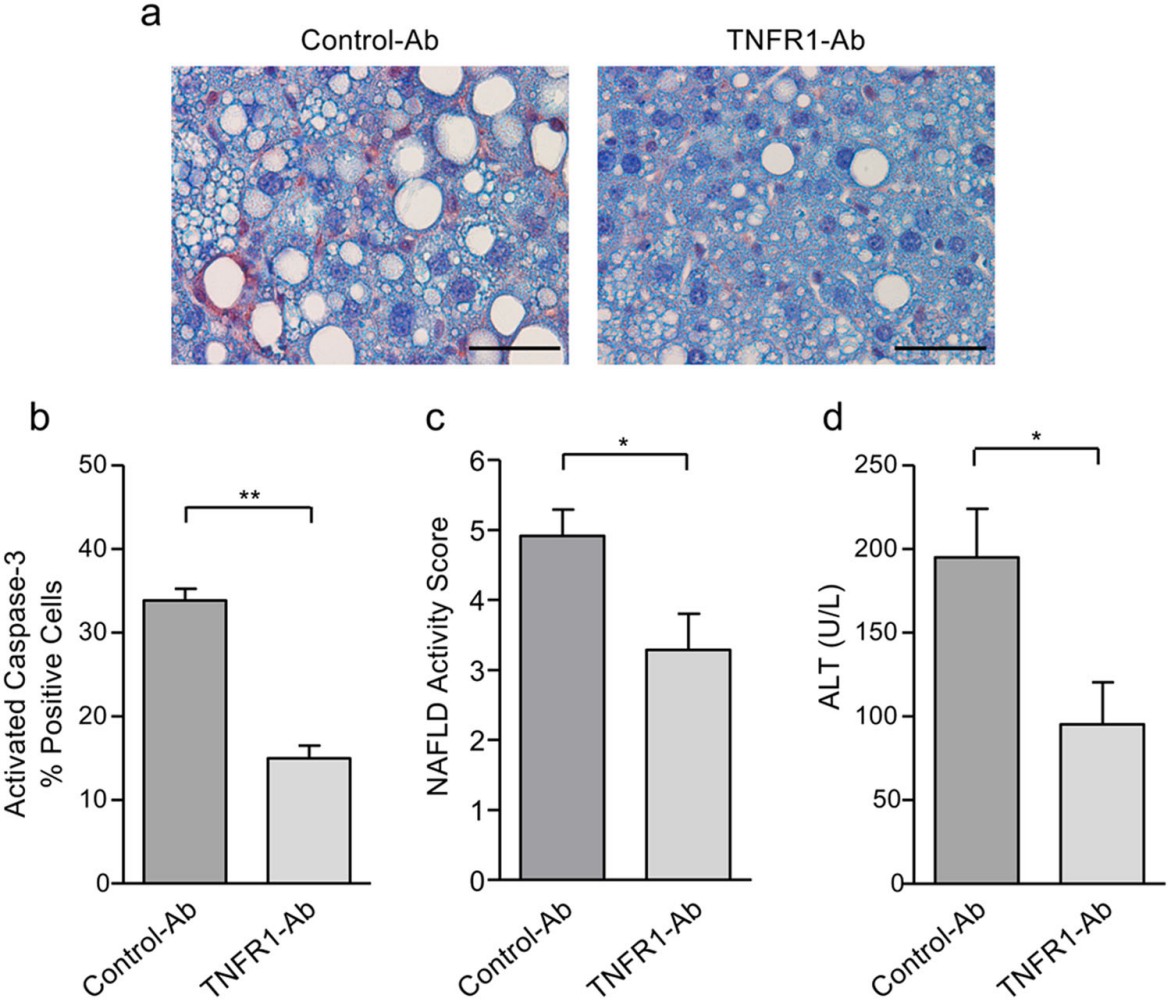
TNFR1 antibody treatment results in reduced liver injury in NAFLD mice. **a** Immunohistochemical detection of activated caspase-3 revealed lower immunoreactivity in liver tissues from NAFLD mice treated with TNFR1-Ab compared to control-Ab-treated mice (scale bars: 50 µm). **b** Caspase-3 activation was significantly decreased in liver tissues from anti-TNFR1-treated (*n* = 7) compared to control-Ab-treated (*n* = 6) NAFLD mice. Percentage of caspase-3 positive cells was assessed by analyzing four microscopic fields at a ×400 magnification and is given as mean ± SEM. **c** NAFLD activity score, which was assessed by a pathologist, was significantly decreased in anti-TNFR1-treated compared to control-Ab-treated mice. **d** Compared to control-Ab treatment, TNFR1 inhibition resulted in a significant decline of alanine aminotransferase (ALT) levels. *p < 0.05; **p < 0.01.

We next investigated the effect of TNFR1 inhibition on liver injury. We analyzed apoptotic liver injury by immunohistochemical detection of activated caspase-3. In line with the reduction of liver steatosis, caspase-3 activity was decreased in liver tissues from anti-TNFR1-treated compared to control-Ab-treated NAFLD mice (Fig. 2a). Quantitative evaluation revealed that the percentage of caspase-3-positive cells was significantly (*p* < 0.01) reduced by anti-TNFR1 compared to control-Ab treatment of NAFLD mice (15.0 ± 1.6% vs. 33.9 ± 1.4%; Fig. 2b). We have also assessed the NAFLD activity score (NAS), which considers the extent of liver steatosis, inflammation and hepatocellular ballooning as a histological sign of liver injury^30^. We found a significant (*p* < 0.05) decrease in the NAS of anti-TNFR1-treated compared to control-Ab-treated mice (3.29 ± 0.52 vs. 4.92 ± 0.37; Fig. 2c). Moreover, all three NAS components declined by anti-TNFR1 compared to control-Ab treatment which was significant for steatosis (data not shown). In line with the improved histological signs of liver injury, ALT levels significantly (*p* < 0.05) decreased with anti-TNFR1 compared to control-Ab treatment (95.3 ± 25.0 U/L vs. 195.0 ± 29.0 U/L; Fig. 2d).

**Fig. 3:**
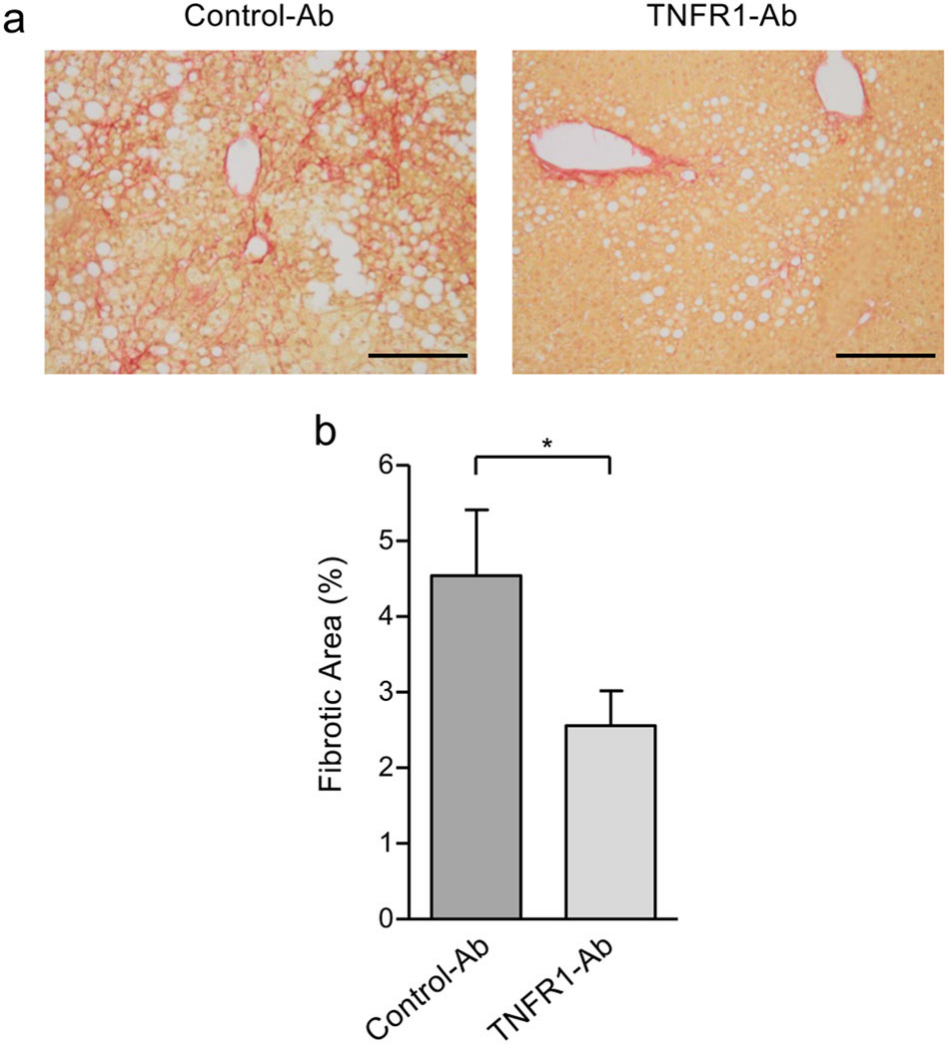
Reduction of liver fibrosis by TNFR1 inhibition in NAFLD mice. **a** Fibrosis, detected by Sirius Red staining, was decreased in TNFR1-Ab-treated compared to control-Ab-treated NAFLD mice (scale bars: 200 µm). **b** Percentage of fibrosis was assessed by analyzing four microscopic fields at ×100 magnification and is given as mean ± SEM. The percentage of fibrosis significantly declined in NAFLD mice treated with TNFR1-Ab (*n* = 7) compared to those treated with control-Ab (*n* = 6); *p < 0.05.

Since apoptotic liver injury triggers fibrosis, which is a major factor of mortality in NAFLD and thus represents an important end point in clinical NAFLD trials, we also assessed the potential role of TNFR1 inhibition in liver fibrosis. The extent of fibrosis, analyzed by Sirius Red staining, was clearly reduced in anti-TNFR1-treated compared to control-Ab-treated NAFLD mice (Fig. 3a). Quantitative analyses revealed a significant (*p* < 0.05) reduction of the fibrotic area in liver tissues from anti-TNFR1-treated mice compared to controls (2.6 ± 0.5% vs. 4.5 ± 0.9%; Fig. 3b). Thus, TNFR1 inhibition not only ameliorates liver injury but also contributes to fibrosis reduction.

### Lower increase of body weight and improved insulin resistance in NAFLD mice treated with anti-TNFR1 antibody

**Fig. 4:**
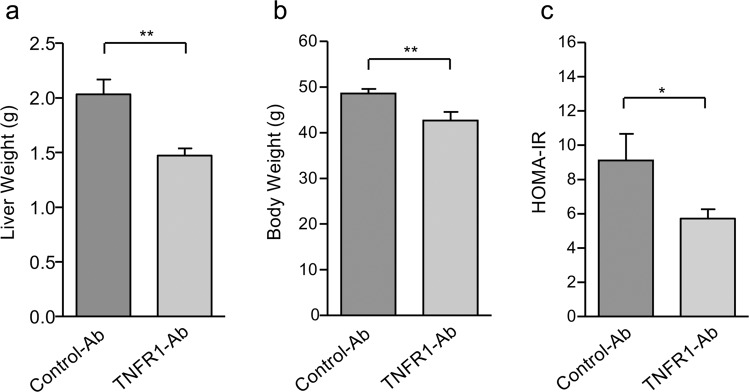
Lower increase of liver and body weight, as well as improved insulin resistance by TNFR1 inhibition in NAFLD mice. **a** NAFLD mice treated with TNFR1-Ab (*n* = 7) revealed a significantly lower liver weight compared to control-Ab-treated (*n* = 6) mice. **b** Also, the increase of body weight was significantly lower in TNFR1-Ab-treated compared to control-Ab-treated mice. **c** Insulin resistance, assessed by HOMA-IR, significantly declined in NAFLD mice treated with TNFR1-Ab compared to control-Ab-treated mice. HOMA-IR, homeostasis model assessment of insulin resistance; *p < 0.05; **p < 0.01.

In addition to a markedly reduced liver steatosis (Fig. 1), TNFR1 inhibition also resulted in a significantly (*p* < 0.01) lower liver weight when compared to control-Ab treatment (1.47 ± 0.07 g vs. 2.03 ± 0.14 g; Fig. 4a). Anti-TNFR1-treated mice revealed also a significantly (*p* < 0.01) lower increase of body weight at the end of treatment compared to control-Ab-treated mice (42.7 ± 1.9 g vs. 48.6 ± 1.0 g; Fig. 4b), which might be caused by metabolic improvement. Indeed, anti-TNFR1 treatment significantly (*p* < 0.05) improved insulin resistance [HOMA-IR 5.7 ± 0.5 (TNFR1-Ab) vs. 9.1 ± 1.6 (control-Ab); Fig. 4c].

**Fig. 5:**
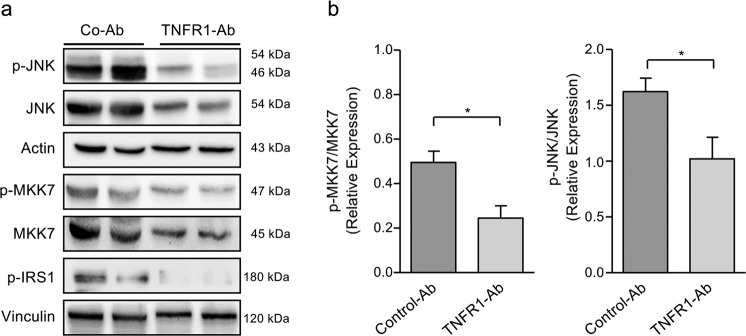
Reduced activation of the MAPK pathway by TNFR1 inhibition in NAFLD mice. **a** Western blot analysis of (p-)JNK, (p-)MKK7 and p-IRS1 in liver tissues from NAFLD mice either treated with TNFR1-Ab or control-Ab. Control-Ab-treated mice showed higher levels of phosphorylated and hence activated JNK, MKK7 and IRS1 in liver tissues compared to TNFR1-Ab-treated mice. **b** Densitometric analysis revealed significantly lower levels of phosphorylated vs. non-phosphorylated forms of JNK and MKK7 in TNFR1-Ab-treated (*n* = 6) compared to control-Ab-treated (*n* = 6) mice. All proteins were detected on the same immunoblot membrane using actin or vinculin as loading control. IRS1, insulin receptor substrate-1; JNK, c-Jun-N-terminal kinase; *p < 0.05.

To further unravel the potential mechanisms of TNFR1 inhibition on insulin resistance we analyzed the activation of JNK and its upstream regulator, the MAP-kinase MKK7, since JNK has been identified as negative regulator of insulin signaling^42,43^. We found an increased MKK7 and JNK activation in liver tissues from NAFLD mice, which was remarkably reduced by anti-TNFR1 but not by control-Ab treatment (Fig. 5a). Furthermore, JNK activation was associated with an increased phosphorylation of the insulin receptor substrate-1 (IRS1), which is known to promote insulin resistance. Anti-TNFR1-treated NAFLD mice revealed strongly reduced IRS1 phosphorylation which might contribute to the improved insulin resistance observed in these mice (Fig. 5a). The slightly reduced expression of non-phosphorylated MKK7 and JNK in liver tissues from anti-TNFR1-treated compared to control mice might be explained by reduced infiltration of immune cells which can also express pro-inflammatory kinases^44^. Densitometric analyses revealed significantly increased expression of phosphorylated in relation to non-phosphorylated MKK7 and JNK in liver tissues from control-treated (*n* = 6) compared to anti-TNFR1-Ab-treated (*n* = 6) mice (Fig. 5b).

## Discussion

TNFα plays a central role in the pathogenesis of NAFLD^4–8^. Binding of TNFα to TNFR1 triggers the activation of the pro-inflammatory MAPK pathway which can result in the activation of JNK^14^. There is increasing evidence that JNK activation contributes to NAFLD progression. Enhanced JNK activation could be demonstrated in murine steatohepatitis and correlated with liver steatosis and inflammation^45,46^. The molecular mechanisms leading to increased JNK activation in NAFLD remain largely unclear. In this study, we demonstrated increased activation of JNK, as well as of its upstream regulator MKK7 in liver tissues from NAFLD mice which could be markedly reduced by TNFR1 inhibition. These data therefore imply an important role of the TNFR1 pathway for MAPK activation in NAFLD.

Previous studies demonstrated that JNK1 mediates Ser307 phosphorylation of IRS1 which blocks its interaction with the insulin receptor, thereby promoting insulin resistance^42,47^. Vice versa, loss of JNK1 prevented the development of insulin resistance and steatohepatitis in various NAFLD mouse models^43,45,46^. In our study we showed that treatment of NAFLD mice with the TNFR1 antibody indeed leads to a significant improvement of insulin resistance which was associated with a decreased Ser307 phosphorylation of IRS1 in the liver. These data therefore suggest that TNFR1-mediated JNK activation and associated phosphorylation of IRS1 critically contribute to insulin resistance in NAFLD. A remarkable observation in this context is that TNFR1-mediated activation of JNK, as well as of the oncogenic transcription factor STAT3 and associated IL-6 production promote obesity-related hepatocarcinogenesis, which is also strongly reduced in TNFR1-deficient mice^22^.

Although lipogenesis is regulated by insulin signaling, it can also be triggered in the insulin-resistant state by the transcription factor SREBP1^48,49^. In this situation, the SREBP1 pathway is activated by mTORC1. Both the expression and activation of SREBP1 are promoted by mTORC1 via different mechanisms involving p70S6 kinase or the phosphatidic acid phosphatase lipin-1^40,41^. The essential role of mTORC1 in lipogenesis is underlined by the observation that SREBP1 activation is prevented by the mTOR inhibitor rapamycin and that mTOR-deficient mice are protected from HFD-induced liver steatosis^39–41^. Enhanced mTOR activation might be caused by AMP-activated protein kinase (AMPK) which normally inhibits mTORC1 but is repressed under nutrient-rich conditions^50^. In this context it is interesting to note that mTOR is a negative regulator of autophagy and that mice deficient for autophagy-regulating genes develop liver steatosis^51,52^. Vice versa, pharmacological autophagy induction reduced liver steatosis and injury in NAFLD mice^53^.

In our study, we found enhanced Ser2448 phosphorylation of mTOR in liver tissues from NAFLD mice which could be reduced by anti-TNFR1 treatment. Whether TNFR1 inhibition and a subsequent reduction of mTOR activation also increase autophagy remains to be investigated. Nevertheless, TNFR1 inhibition reduced SREBP1 activation and downstream targets of lipogenesis such as FAS and SCD1, resulting in reduced liver triglyceride content and steatosis. SREBP1 not only plays a role in lipogenesis but can also transcriptionally repress IRS2 which contributes to insulin resistance^54^. In this context, increased SREBP1 activity was found to be associated with decreased hepatic IRS2 expression and hyperinsulinemia in obese diabetic mice^55^. Intriguingly, we found enhanced IRS2 mRNA expression in liver tissues from anti-TNFR1-treated compared to control-Ab-treated NAFLD mice (data not shown).

There is increasing evidence that lipid accumulation triggers hepatocyte apoptosis which plays an important role for NAFLD activity and progression. For instance, free fatty acids induce oxidative stress which leads to mitochondrial dysfunction, caspase activation and apoptotic cell death^19^. In addition to the mitochondrial pathway, apoptosis can be induced by death receptors such as CD95, TRAIL-R1/2 or TNFR1 in NAFLD^17^. It is also interesting to note that TNFα-induced hepatocyte apoptosis involves JNK-mediated activation of pro-apoptotic molecules such as Bim^56^. In this study, we demonstrated increased caspase-3 activation in liver tissues from HFD-fed mice, which was markedly reduced by the anti-TNFR1 antibody. Furthermore, anti-TNFR1-treated NAFLD mice revealed a decline of the NAFLD activity score in liver tissues compared to control-Ab-treated mice. The reduced liver injury was accompanied by significantly lower ALT levels in anti-TNFR1-treated compared to control-Ab-treated NAFLD mice. These data indicate that TNFR1 inhibition not only reduces insulin resistance and lipid accumulation but also ameliorates liver injury. An interesting finding is that active caspases can cleave SREBP1 and might therefore contribute to its lipogenic activity, which could explain the observation that caspase activity correlates with liver steatosis^57,58^.

Apoptotic cell death contributes to liver fibrosis which represents a major prognostic factor in NAFLD^3^. In support of this idea, it was shown that engulfment of apoptotic bodies by stellate cells stimulates their fibrogenic activity, whereas inhibition of hepatocyte apoptosis reduced liver injury and fibrosis in a murine NASH model^59,60^. Strikingly, we could demonstrate that TNFR1 inhibition significantly decreased liver fibrosis in NAFLD mice. The role of TNFR1 for fibrogenesis is supported by the observation that this receptor is required for the proliferation and fibrogenic activity of hepatic stellate cells^20^.

Taken together, our study implicates an important role of TNFR1 signaling for the development of insulin resistance and fibrotic liver injury in NAFLD, which both are of prognostic relevance for this disease. Inhibition of TNFR1 might therefore represent a promising approach for NASH treatment. These findings could provide the basis for future clinical trials analyzing the potential therapeutic efficiency of TNFR1 inhibition in NASH patients.
